# Vitamin D Endocrine System and COVID-19: Treatment with Calcifediol

**DOI:** 10.3390/nu14132716

**Published:** 2022-06-29

**Authors:** Jose Manuel Quesada-Gomez, José Lopez-Miranda, Marta Entrenas-Castillo, Antonio Casado-Díaz, Xavier Nogues y Solans, José Luis Mansur, Roger Bouillon

**Affiliations:** 1 Instituto Maimónides de Investigación Biomédica de Córdoba (IMIBIC), Hospital Universitario Reina Sofía, 14004 Córdoba, Spain; md1lomij@uco.es (J.L.-M.); bb1cadia@uco.es (A.C.-D.); 2 Centro de Investigación Biomédica en Red de Fragilidad y Envejecimiento Saludable (CIBERFES), Instituto de Salud Carlos III, 28029 Madrid, Spain; xnogues@psmar.cat; 3 Departamento de Medicina Interna, Hospital Universitario Reina Sofía, 14004 Córdoba, Spain; 4 CIBER Fisiopatologia Obesidad y Nutrición (CIBEROBN), Instituto de Salud Carlos III, 28029 Madrid, Spain; 5 Hospital QuirónSalud, 14004 Córdoba, Spain; marenca@gmail.com; 6 Unidad de Gestión Clínica de Endocrinología y Nutrición, Hospital Universitario Reina Sofía, 14004 Córdoba, Spain; 7 Internal Medicine Department, IMIM (Hospital del Mar Medical Research Institute), Hospital del Mar, 08003 Barcelona, Spain; 8 Centro de Endocrinología y Osteoporosis La Plata, Buenos Aires B1902ADQ, Argentina; joseluismansur@yahoo.com.ar; 9 Clinical and Experimental Endocrinology, Department of Chronic Diseases and Metabolism, Catholic University of Leuven, 3000 Leuven, Belgium

**Keywords:** calcifediol, calcitriol, cholecalciferol, COVID-19, SARS-CoV-2, vitamin D endocrine system

## Abstract

The COVID-19 pandemic is the greatest challenge facing modern medicine and public health systems. The viral evolution of SARS-CoV-2, with the emergence of new variants with in-creased infectious potential, is a cause for concern. In addition, vaccination coverage remains in-sufficient worldwide. Therefore, there is a need to develop new therapeutic options, and/or to optimize the repositioning of drugs approved for other indications for COVID-19. This may include the use of calcifediol, the prohormone of the vitamin D endocrine system (VDES) as it may have potential useful effects for the treatment of COVID-19. We review the aspects associating COVID-19 with VDES and the potential use of calcifediol in COVID-19. VDES/VDR stimulation may enhance innate antiviral effector mechanisms, facilitating the induction of antimicrobial peptides/autophagy, with a critical modulatory role in the subsequent host reactive hyperinflammatory phase during COVID-19: By decreasing the cytokine/chemokine storm, regulating the renin–angiotensin–bradykinin system (RAAS), modulating neutrophil activity and maintaining the integrity of the pulmonary epithelial barrier, stimulating epithelial repair, and directly and indirectly decreasing the increased coagulability and prothrombotic tendency associated with severe COVID-19 and its complications. Available evidence suggests that VDES/VDR stimulation, while maintaining optimal serum 25OHD status, in patients with SARS-CoV-2 infection may significantly reduce the risk of acute respiratory distress syndrome (ARDS) and severe COVID-19, with possible beneficial effects on the need for mechanical ventilation and/or intensive care unit (ICU) admission, as well as deaths in the course of the disease. The pharmacokinetic and functional characteristics of calcifediol give it superiority in rapidly optimizing 25OHD levels in COVID-19. A pilot study and several observational intervention studies using high doses of calcifediol (0.532 mg on day 1 and 0.266 mg on days 3, 7, 14, 21, and 28) dramatically decreased the need for ICU admission and the mortality rate. We, therefore, propose to use calcifediol at the doses described for the rapid correction of 25OHD deficiency in all patients in the early stages of COVID-19, in association, if necessary, with the new oral antiviral agents.

## 1. Introduction

The COVID-19 pandemic, or SARS-CoV-2 coronavirus disease, is the greatest challenge facing modern medicine and public health systems worldwide [1]. Entering the third year of the global COVID-19 pandemic, since it was first reported in December 2019 in Wuhan (China) until 15 March 2022, it has caused worldwide a total of 460,844,076 confirmed cases and a total of 6,069,430 deaths [2]. Besides, the total impact of the pandemic has been far greater than the reported deaths from COVID-19. Despite the global implementation of hygienic preventive measures (e.g., social distancing, confinements, use of masks, frequent hand washing) and large-scale vaccination programs in all countries of the world, global morbidity and mortality due to COVID-19 remain high [3].

The viral evolution of SARS-CoV-2, with the emergence of new variants with increased infectious potential [4], such as Omicron (Pango lineage B.1.1. 529), which contains 15 mutations in the receptor binding domain (RBD), are cause for concern. Besides, the efficacy of currently available vaccines against these viral mutants may be lower, mostly related to short-term immunity [5], in addition, vaccination coverage remains insufficient worldwide [6]. Therefore, it is desirable that we continue to deepen our knowledge of the immune response to SARS-CoV-2, to improve our understanding of the pathogenesis of COVID-19. It is necessary to develop new therapeutic options and/or optimize the repositioning of drugs already approved for use in humans for another indications [7], such as the use of calcifediol, a prohormone of the vitamin D endocrine system (VDES), which has potential useful actions for the treatment of COVID-19 [8,9], which we review hereafter.

## 2. COVID-19 (Coronavirus Disease 2019)

The coronavirus, SARS-CoV-2 (severe acute respiratory syndrome coronavirus 2), is responsible for COVID-19 (coronavirus disease 2019). The analysis of the coronavirus genome has revealed that the sequence of SARS-CoV-2 is 79.5–82.0%, identical to that of SARS-CoV [10] Its genome encodes for a number of viral structural proteins, the spike glycoprotein (S), envelope protein (E), membrane protein (M), nucleocapsid protein (N), and nine accessory proteins (Orf3a, Orf3b, Orf6, Orf7a, Orf7b, Orf8, Orf9b, Orf9c, and Orf10) [11,12]. The S protein is responsible for viral entry into host cells by direct receptor binding and fusion with the host cell membrane [13]. 

**Figure 1:**
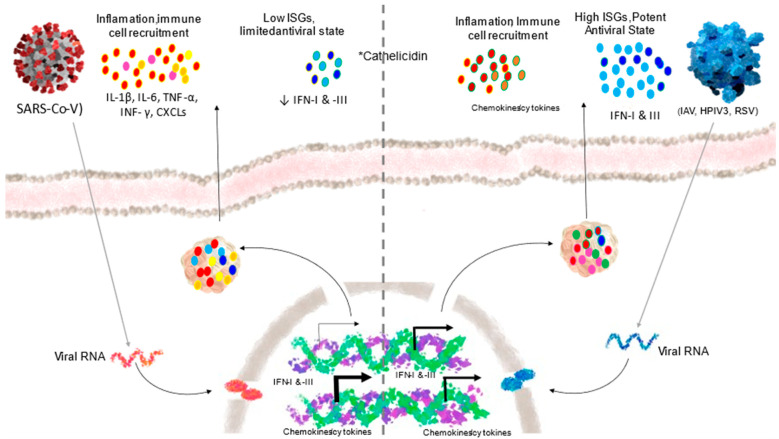
COVID-19 is characterized by an unbalanced host response to SARS-CoV-2 following viral replication, which depending on its intensity will characterize the development and severity of COVID-19: (1) reduction in innate antiviral defenses; (2) exuberant production of inflammatory cytokines, with inadequate recruitment of inflammatory populations of monocytes and macrophages. Comparison with other viral infections IL: interleukin; IFN: interferon; ISG: interferon-stimulated genes; TNF: tumor necrosis factor; CXCLS: chemokine (C-X-C motif) ligands; IAV: influenza A virus; HPIV3: human parainfluenza virus type 3; RSV: respiratory syncytial virus. * When calcifediol levels are deficient, the cathelicidin response is impaired. Modified from Blanco Melo et al. [18].

The clinical spectrum of COVID-19 is variable [14]. Epidemiological studies have reported that the vast majority (80%) of patients infected with SARS-CoV-2 during the acute phase of infection are asymptomatic or show mild symptoms; approximately 20% progress to severe symptomatology, of which 5% develop acute respiratory distress syndrome (ARDS), septic shock, and multi-organ failure accompanied by a high risk of death [15,16]. Some patients continue to have long-term symptoms, termed post-acute sequelae of SARS-CoV-2 or long-COVID, a condition in which COVID-19 symptomatology persists beyond 3–4 weeks after initial infection, with pulmonary, cardiovascular, hematological, neuropsychiatric, renal, endocrine, gastrointestinal, hepatobiliary, and inflammatory sequelae [17]. The natural history of COVID, and the evolution of SARS-CoV-2 infection, is conditioned by cell tropism and host immune response [18] (Figure 1).

## 3. Vitamin D Endocrine System—Calcifediol

Vitamin D generates many metabolites and “vitamin D” is frequently ambiguously used as cholecalciferol (vitamin D3), ergocalciferol (vitamin D2), calcifediol (25 hydroxyvitamin D3), and calcitriol (1,25-dihydroxyvitamin D3) and sometimes even their analogues [19,20,21].

**Figure 2:**
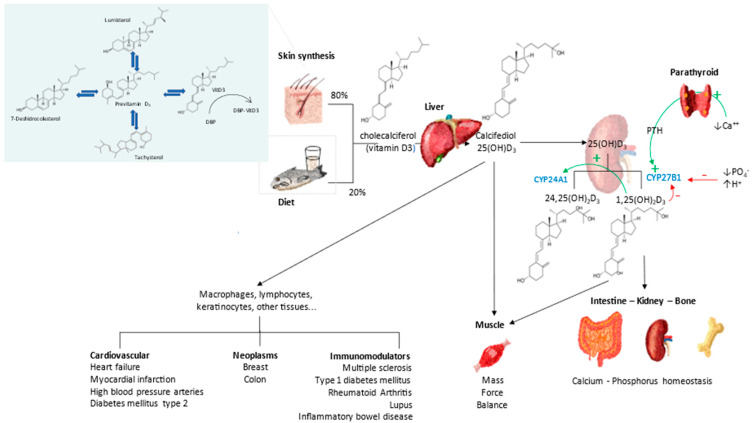
Vitamin D Endocrine system. Metabolism and actions.

Since its discovery just over a century ago, we now know that it is not a vitamin, but a threshold nutrient and part of the vitamin D endocrine system (VDES), similar to other steroid hormones [22,23] (Figure 2).

“Vitamin” D3 is totally inactive, requiring two sequential hydroxylations, at the 25- and 1α-position to become active. The first in the liver is mainly by the microsomal enzyme CYP2R1 to form 25-hydroxyvitamin D (25OHD3) or calcifediol, and a second hydroxylation (CYP27B1) is mainly in the proximal tubule of the kidney, but also in many other cell types (i.e., skin, immune cells, lung, bone cells, placenta etc.) to obtain the active metabolite, i.e., 1,25-dihydroxyvitamin D3 (1,25(OH)2D3) or calcitriol, the VDES hormone [24]. Vitamin D3, whether synthesized in the epidermis or from dietary sources, is rapidly taken up (half of an oral dose is taken up in 2–6 h), principally by the liver, but also by other tissues, such as adipose and muscle. Hydroxylation to synthesize 25OHD3 occurs mainly in the liver, although other tissues express this enzymatic activity as well [24].

Calcifediol (25OHD3) is the prohormone and cornerstone of the VDES. It is the metabolite with the highest blood concentration and longest half-life (2–3 weeks). There is universal agreement that the measurement of the total 25OHD blood concentration is the marker of the nutritional status of the vitamin D endocrine system [25]. Its measurement has been used by health authorities and scientific societies to establish normal status, define vitamin D deficiency and degrees of vitamin D insufficiency, on which to establish vitamin D reference intake values, as well as to perform population monitoring of vitamin D deficiency, insufficiency, or excess [26,27].

1,25(OH)2D is the hormone of such endocrine system, having a short half-life (5–8 h). It is produced from its precursor 25OHD, by the enzymatic activity of 25OHD-1α hydroxylase. It is carried out in kidney tubular cells for its endocrine actions, tightly regulated by parathyroid hormone (PTH), fibroblast growth-factor 23 (FGF23), calcium, phosphate, and 1,25(OH)2 D. Likewise in epidermal keratinocytes testes, intestine, lung, breast, macrophages, activated lymphocytes, parathyroid gland, osteoblasts, and chondrocytes, for their local actions (autocrine and paracrine), with less tight regulation. The extrarenal synthesis of 1,25(OH)2D is stimulated by cytokines, such as interferon gamma and TNF-α [24].

Calcitriol binds with high affinity to the vitamin D receptor (VDR), a nuclear transcription factor present in the cells of multiple organs and systems, which determines the systemic endocrine and auto/paracrine action of the VDES [28]. The classical VDR belongs to the nuclear receptor superfamily. Ligand binding results in heterodimerization with the retinoic X receptor (RXR [28]). Most cells respond to VDR activation by changes in gene expression, protein synthesis, or cell differentiation and function [24,28].

The main action of VDES is the regulation of calcium and phosphorus homeostasis and the adequate health and mineralization of the skeleton. However, experimental animal, cellular, molecular, and genetic studies consistently suggest that VDR signaling has numerous extra-osseous actions. These include muscle and immune function, skin differentiation, regulation of cell proliferation, and aspects of reproduction, as well as metabolic and cardiovascular properties. Based on observational studies in patients, 25OHD deficiency has been associated with almost all of the diseases predicted by these extraosseous effects [24,28].

During evolution, the immune system and the VDES had an interesting synchronous development, whereby cells of the immune system are a target of the VDES, and calcitriol regulates metabolic signaling pathways and multiple crucial cytokines of the immune system (innate and adaptive) [29,30]. The localized synthesis of calcitriol from calcifediol in alveolar macrophages, dendritic cells, lymphocytes [31], as well as in broncho alveolar cell epithelium and pulmonary endothelial cells, may be responsible, in an autocrine or paracrine manner, for many of the immunological and extraosseous effects of VDES [28].

Other actions include the stimulation of proliferation, alveolar cell differentiation and the expression of some essential lung genes (including surfactant protein) [32]. VDES is also involved in the functional regulation of the cardiovascular system [33,34] and is clearly involved via several pathways in coagulation mechanisms [35].

The upper and lower airway epithelium and the immune and cardiovascular systems, which play a key role in COVID-19 [36], are targets of VDES. The 25OHD/calcifediol deficiency is highly prevalent worldwide [37,38] and has been associated with a number of diseases [39], including immune disorders, pulmonary, infectious, and cardiovascular diseases [40], as well as idiopathic deep vein thrombosis of the lower extremities [41].

It is, therefore, not surprising that since the beginning of the COVID-19 pandemic, a possible link between a deficient 25OHD status and COVID-19 infection and/or COVID-19 severity has been proposed. This, from the beginning of this pandemic until now, has generated more than 1100 publications listed in PubMed associating the keywords “COVID-19” and “vitamin D”.

We now have strong consistent evidence that VDES dysregulation in the lung and immune cells of SARS-CoV-2 infected patients [42], and that VDR stimulation could enhance the antiviral response and reduce cytokine storm [43]. Another study on the systematic reuse of potential drugs to be used in COVID-19, based on machine learning, has reported that VDR stimulation could have a protective effect on pathways affected by SARS-CoV-2 infection [7], suggesting a potential protective role of VDES metabolites in the treatment of COVID-19.

In the following, we will firstly review the available data on possible mechanisms by which VDES may protect against COVID-19 or its complications. Secondly, we will summarize the clinical data (observational and interventional studies) linking calcifediol to COVID-19.

## 4. Mechanisms Linking Vitamin D Endocrine System and COVID-19

Several studies, prior to COVID-19, link serum 25OHD status and upper respiratory tract infections [9,44]. Observational studies reported an increased risk of infections in patients with low serum 25OHD levels [45,46], and similarly, certain vitamin D receptor (VDR) polymorphisms have been associated with increased risk of acute lower respiratory tract infections [46].

Recently, two meta-analyses of randomized controlled trials (albeit heterogeneous) conducted between 2007 and 2020 show a significant reduction in the risk of upper respiratory tract infection and daily or weekly vitamin D supplementation, especially when the baseline level prior to study entry is low [47,48].

The regulatory effects of VDES in optimizing innate and adaptive immune function have been rigorously reviewed by Greiller and Martineau as well as others [45,49,50], and several recent reviews pick up on the mechanisms of interaction between VDES and SARS-CoV-2 infection [9,51,52,53]. VDES/VDR signaling may provide beneficial effects on COVID-19 by several mechanisms.

### 4.1. Vitamin Endocrine D System and the Immune System

#### 4.1.1. Innate Immunity

Respiratory monocytes/macrophages, bronchial epithelial cells, and type II alveolar cuboidal lining cells (ACII) constitutively express the gene encoding VDR, with high basal expression of 1α-hydroxylase (CYP27B1) and low expression of the genes encoding 24-hydroxylase (CYP24A1) and other VDES proteins. The genes encoding the β-defensins and LL-37 contain consensus vitamin D response elements (VREs) [24,28].

**Figure 3:**
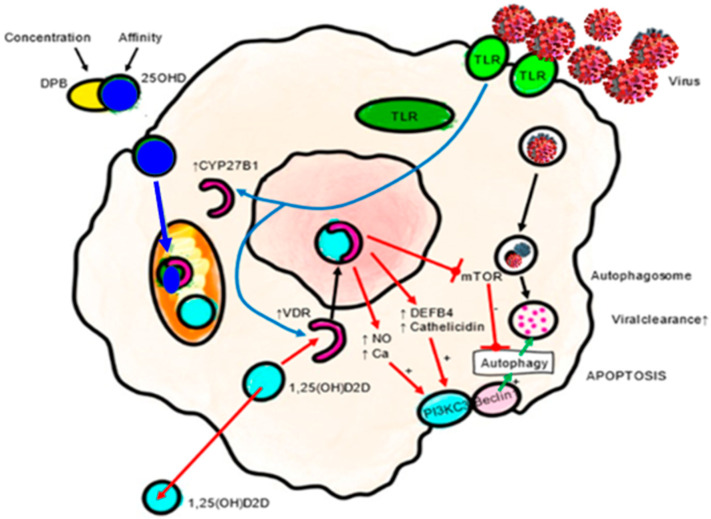
Antiviral actions of VDES and the innate immune response: autophagy/apoptosis. Autophagy is an essential mechanism by which cells cope with viruses. Autophagic encapsulation of viral particles packages them for lysosomal degradation and subsequent presentation of antigens and adaptive antiviral immune responses. Therefore, autophagy may be highly sensitive to changes in 25OHD serum levels. The specific mechanisms by which VDES promotes autophagy involve down regulation of the mTOR pathway, which inhibits autophagy, and the promotion of Beclin 1 and PI3KC3, key autophagy-driving enzymes. The upregulation of intracellular Ca and NO by VDES also stimulates the activity of PI3KC3 to promote autophagy. DEFB4A: defensin beta 4A. mTOR: mammalian target of rapamycin. Ca: calcium. NO: nitric oxide. PIK3C3: phosphatidylinositol 3-kinase catalytic subunit type 3. TLR: toll-like receptor. VDR: vitamin D receptor.

The intracrine activation of the VDES include induction of the AMP cathelicidin (cAMP) and β-defensin (DEFB4), as well as modulation of autophagy. That results in enhanced defense against viral infections [54]. Cathelicidin not only has potent prophylactic and therapeutic potential in COVID-19 as an inhibitor of viral binding to ACE2, but also modulates local inflammation and leukocyte migration and infiltration, reducing the production of proinflammatory cytokines and chemokines in acute lung injury. Furthermore, it activates the expression of interferon I, which is critical in SARS-CoV-2 infection, all of which is beneficial in mitigating the cytokine storm that follows SARS-CoV-2 infection (Figure 3).

Calcitriol also promotes an antioxidant effect in monocytes by up-regulating glutathione reductase (GR) and glutamate–cysteine ligase (GCL), which reduces the production of oxygen radicals [55]. It can also stimulate viral autophagy [56,57]. VDR activation can also inhibit S-phase kinase-associated protein 2 (Skp2), which plays a key role in the viral replication mechanism in COVID-19 [58].

The limiting element of this defense mechanism against viruses and bacteria is that it requires the adequate availability of calcifediol at the time of infection, and serum levels of 25OHD may vary widely, even within populations [23].

Epithelial barrier is the first line of defense for physically protecting the host against bacterial, fungal, viral, and parasitic pathogens. VDR stimulation plays an important role in maintaining epithelial barrier homeostasis and integrity in multiple organs [59] by preserving the integrity of junctional complexes [60]. The same has been observed in the lung epithelium, where VDR deletion leads to the destruction of tight and adherents junction proteins (such as claudins ZO-1, occludin, etc.) resulting in reduced tight junctions and compromised lung barrier integrity [61]. 

These data indicate that VDES has therapeutic potential for contributing to the prevention or resolution of ARDS, which is associated with significant damage to the alveolar epithelial barrier.

#### 4.1.2. Adaptive Immunity

Cytokine and chemokine storm is one of the most devastating pathophysiological aspects of SARS-CoV-2 infection and is a major cause of morbidity and mortality. It is an exaggerated activation of the adaptive immune pathway, with an exuberant secretion of pro-inflammatory cytokines and chemokines because of dysregulation of the innate immune system [18,62]. 

Calcitriol, produced locally from calcifediol by bronchoalveolar epithelial cells, monocytes/macrophages and activated lymphocytes, can dramatically change the immune status from a proinflammatory to a tolerogenic state, suppress T-lymphocyte proliferation and modulate cytokine production and differentiation with diverse effects on different T-lymphocyte subsets [63], and may contribute to minimizing the COVID-19 cytokine and chemokine crisis. Calcitriol results in anti-inflammatory activity on macrophages by increasing interleukin (IL)-10 and decreasing inflammatory stimuli [64].

Calcitriol drives antigen-presenting dendritic cells (DC) towards a less mature and more tolerogenic phenotype, as evidenced by morphological change and altered cytokine production and changes in surface marker expression. 

**Figure 4:**
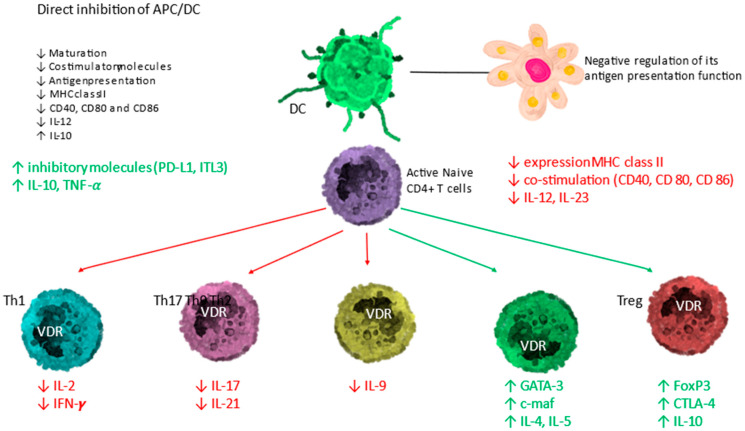
Immunomodulatory activity of the vitamin D endocrine system. Activated DC and lymphocytes have the ability to form calcitriol from circulating calcifediol. The calcitriol formed exerts effects through VDR on antigen-presenting cells (APC)/dendritic cell (DC) and T lymphocytes. The effect is an upward regulation of direct inhibition of DC and a downward regulation of antigen presentation. On T lymphocytes, the direct effect consists of an induction of T helper-2 lymphocytes (Th2) and regulatory T lymphocytes (Tregs) (green arrows) represented in green text, together with a downward regulation of T helper-1 (Th1), T helper-17 (Th17)-lymphocytes and T helper-9 (Th9)-lymphocytes (red arrows).

Calcitriol shifts the balance of the adaptive immune system from Th1, Th9, and Th17 lymphocytes to the Th2 and regulatory T lymphocytes (Tregs) immune profile, by suppressing the expression of Th1 (IL-2, IFN-γ and TNF-α), Th9 (IL-9), and Th17 (IL-17 and IL-21) cytokines while inducing the expression of Th2 cytokines (IL-4, IL-5, IL-9 and IL-13). The global effect is summarized in Figure 4.

The calcitriol formed also promotes the differentiation of regulatory T cells (Treg), both directly and indirectly through their interaction with antigen-presenting cells, contributing to the suppression of the pro-inflammatory state.

APC = antigen-presenting cell; DC = dendritic cell; naïve T cells MHC = membrane histocompatibility complex; cluster of differentiation (CD) 80 = CD86 (co-stimulatory molecules), and CD54 (adhesion molecule); PD-L1 = programmed death-ligand 1; ILT-3 = immunoglobulin-like transcript, T lymphocytes; TH1 = T helper 1; TH2 = T helper 2; TH17 = T helper 17; Treg = regulatory T cell; IL = interleukin; TNF-α = tumor necrosis factor-α; FoxP3 = Forkhead box P3 (master gene controlling the development and function of regulatory cells); CTLA-4 = cytotoxic T lymphocyte-associated Ag-4). Modified from Bouillon R and Quesada-Gomez JM [8].

### 4.2. Vitamin D Endocrine System and Renin–Angiotensin–Aldosterone System (RAAS)

**Figure 5:**
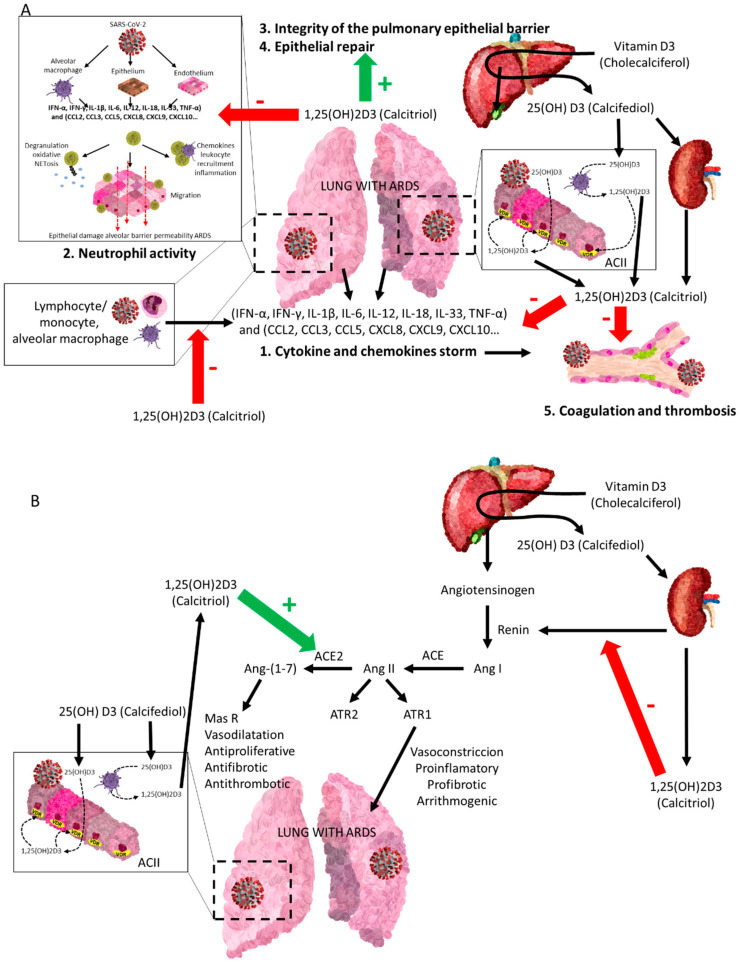
The vitamin D endocrine system (VDES) contributes to the reduction in acute respiratory distress syndrome (ARDS) and related clinics in COVID-19. Vitamin D receptor (VDR) and vitamin D endocrine system enzymes are expressed in activated monocytes/macrophages/granulocytes and lymphocytes and in bronchoalveolar epithelial cells. The availability of 25OHD3 (calcifediol) is essential for synthesizing 1,25(OH)2D3 (calcitriol), which through its endocrine, auto/paracrine action on VDR A: (1) decreases the intensity of the cytokine and chemokine storm, (2) modulates neutrophil activity, (3) maintains the integrity of the pulmonary epithelial barrier, (4) stimulates epithelial repair, and (5) directly and indirectly decreases the risk of hypercoagulability and pulmonary or systemic thrombosis. B: is a powerful negative regulator of the RAAS, inhibiting renin and the ACE/Ang II/AT1R cascade and inducing ACE2/Ang-(1-7) axis activity, contributing to decrease the intensity of ARDS in all its aspects, following SARS-CoV-1 infection. (**A**) SARS-CoV-2 = severe acute respiratory syndrome coronavirus 2; IFN-α, IFN-γ = interferon gamma α and γ; IL-1β, IL-6, IL-12, IL-18, IL-33 = interleukin-1β, 6, 12, 18, 33; TNF-α = tumour necrosis factor-α; TGFβ = transforming growth factor α and β; CCL2, CCL3, CCL5 Chemokine = C-C motif ligand 2, 3, 5; CXCL8, CXCL9, CXCL10 = C-X-C (chemokine motif ligand 8, 9, 10). (**B**) ACII = alveolar cuboidal cells type II; SARS-CoV-2 = severe acute respiratory syndrome coronavirus 2; Ang I = angiotensin I; Ang II = angiotensin II; Ang-(1-7) = angiotensin 1-7; MasR = Mas G-protein-coupled receptor; AT1R and AT2R = angiotensin II receptor 1 and 2.

VDES is a potent negative regulator of RAAS, which is of paramount importance in the development of severe COVID-19, contributing significantly to ARDS and its maintenance. VDR activation negatively regulates ACE1 (and its proinflammatory consequences), but also positively regulates ACE2 by decreasing RAAS activity, both systemically and in the kidney (Figure 5).

In models of LPS-induced respiratory failure, calcitriol has been shown to repress renin, ACE, and Ang II expression, inducing ACE2 expression [65,66]. On the other hand, the dysregulation of local and circulating RAAS, inducing increased ACE/Ang II expression levels, and reduced ACE2/Ang-(1-7) expression levels have been reported to contribute to worsening the course of ischaemia–reperfusion-induced acute lung injury (ALI) [67]. Therefore, VDR stimulation may at least partially attenuate LPS-induced ALI by enhancing ACE2/Ang-(1-7) axis activity and inhibiting renin and the ACE/Ang II/AT1R cascade [66].

### 4.3. Vitamin D Endocrine System and the Coagulation System

The activation of the RAAS, together with intense inflammation, can alter the coagulation cascade. This, combined with infection of endothelial cells results in a prothrombotic state, as found in SARS-CoV-2 infections. Indeed, intra-alveolar or systemic fibrin-clot formation and thrombotic complications are prominent findings in patients with COVID-19.

VDR activation plays an important anti-inflammatory and anti-thrombotic role. Calcitriol (i) inhibits the maturation and activity of dendritic cells and the inflammatory response of effector T cells; (ii) in T/B cells activates anti-inflammatory IL-10 production; (iii) down-regulates IL-6, TNF, NF-κB, and monocyte-chemoattractant-protein-1 (MCP-1); in macrophages activates the antimicrobial peptide cathelicidin; (iv) down-regulates IFNγ, IL-17 and IL-21 in T cells; (v) up-regulates the natural anticoagulants thrombomodulin (TM) and tissue factor pathway inhibitor (TFPI), deactivating tissue factor (TF); and (vi) down-regulates the natural anticoagulants thrombomodulin (TM) and tissue factor pathway inhibitor (TFPI), thereby reducing the hypercoagulable state [68]. These antithrombotic effects have been well documented in VDR-null mice [69,70]. In patients with ischaemic stroke, observational studies reported an association between low 25OHD levels and development of deep-venous thromboembolic events. Furthermore, a significant positive association was found between TFPI (a dual coagulation inhibitor that binds to both the TF/Factor VIIa complex and Factor Xa) and serum 25OHD levels (>20 ng/mL) [71].

### 4.4. Vitamin D Endocrine System and Fibrosis

The activation of the TGF-β signaling pathways in human lung epithelial cells is reduced by calcitriol, which down-regulates fibronectin and collagen expression, thereby inhibiting transdifferentiation of stimulated lung epithelial cells into myofibroblasts [72]. Calcifediol and calcitriol, acting on the local renin–angiotensin system in the lungs, are able to suppress induced pulmonary fibrosis [73,74].

Thus, from a mechanistic perspective, there is good reason to postulate that VDES metabolites, in addition to host responses to ARDS, in the early viral phase (via innate antiviral effector mechanisms, including induction of antimicrobial peptides and autophagy), may have a critical modulatory role in the later hyperinflammatory phase of COVID-19. The activation of the VDR signaling pathway may generate beneficial effects by, decreasing the cytokine/chemokine storm, producing a shift from a Th1 and Th17 phenotype towards adaptive immune responses with an amplified Th2 phenotype; regulating the renin–angiotensin–bradykinin system (RAAS); modulate neutrophil activity and maintain the integrity of the pulmonary epithelial barrier; stimulating epithelial repair and directly and indirectly decreasing the increased coagulability and prothrombotic tendency associated with severe COVID-19 and its complications.

## 5. Circulating 25OHD Levels and Incidence and Severity of COVID-19

Since April 2020, many epidemiological and association studies have been published, investigating the relationship between the circulating levels of 25OHD and outcomes of SARS-CoV-2 infection, related to the incidence, severity, and mortality of COVID-19. Most but not all publications find an association with decreased levels of 25OHD. There are no clear reasons for such discrepancy, but this could be related to the heterogeneity of the patients, disease severity, or the interpretation of severity used by each author at the time the study, as well as the objective of the study (admission, survival, death, need for intensive care unit). Furthermore, most studies are observational and do not correct for various comorbidities. Moreover, most of the studies measured circulating levels of 25OHD at the time of SARS-CoV-2 infection, so the possibility of reverse causality in the reduction in total 25OHD levels cannot be completely ruled out, given the large inflammatory component of the disease.

Several small meta-analyses showed that lower 25OHD levels are associated with increased patient susceptibility to infection, higher rates of hospital admissions, longer hospital stays, increased need for mechanical ventilation or intensive care unit admission, and higher COVID-19 mortality [75,76,77,78,79,80,81,82,83]

A larger meta-analysis included 54 clinical studies, representing a total of 1,403,715 patients concluded that low 25OHD levels are associated with increased risk of SARS-CoV-2 infection, severity (hospitalization and ICU admission), and mortality from COVID-19, regardless of the cut-off point chosen in the assessment (severe deficiency (<10 ng/mL), deficiency (<20 ng/mL), and insufficiency (<30 ng/mL)) [83]. The most recent meta-analysis evaluated studies with more than 2 million subjects and concluded that serum 25OHD levels below 20 ng/mL increased 1.46 fold the risk of being infected by SARS-CoV-2 [84].

## 6. Calcifediol Treatment for COVID-19

The data summarized above suggest a link between the VDES and COVID-19 infections. The observational data also suggest that a poor vitamin D status may aggravate the course of this viral infection. Intervention studies are of course the final proof for causality and efficacy. The vitamin D status can be improved by administration of vitamin D or by intake of calcifediol.

Correcting 25OHD deficiency in critically ill patients by cholecalciferol supplementation requires much higher doses than usual [85]. As an alternative strategy to increase the serum 25(OH)D3 concentrations in vitamin D-deficient adults, oral supplementation of 25(OH)D3 (calcifediol) has been suggested [86,87].

Calcifediol may have some advantages over native vitamin D (cholecalciferol or vitamin D3, and ergocalciferol or vitamin D2) [88], which gives it a certain superiority for use in COVID-19: (1) calcifediol induces a more rapid increase in circulating 25OHD than oral cholecalciferol; (2) oral calcifediol is more potent than cholecalciferol; (3) oral calcifediol has a higher rate of intestinal absorption, which confers advantages in cases of malabsorption; (4) oral calcifediol has a linear dose–response curve, independent of initial serum 25OHD [89].

**Figure 6:**
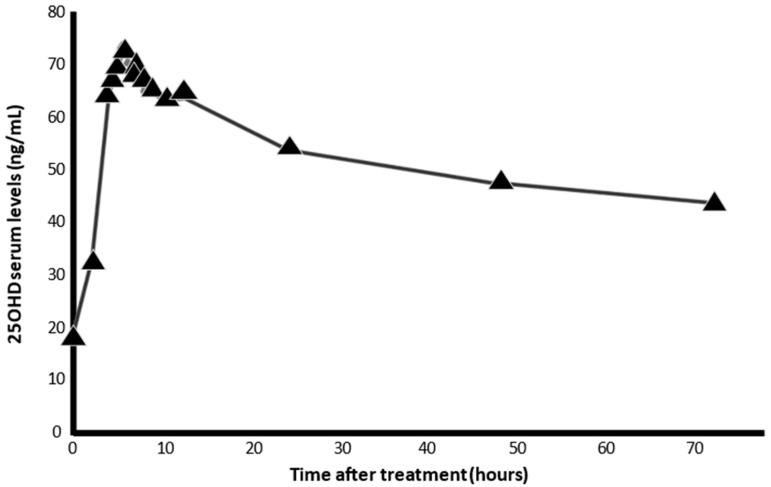
Serum levels of 25OHD, after intake of 0.532 mg calcifediol in soft capsules (AUCu 72 ng/mL). The pharmacokinetic characteristics of calcifediol allow it to be rapidly absorbed within hours, facilitating the immediate availability of 25OHD3 in target tissues (results provided from the technical dossier of the product by FAES-Farma. Lejona. (Spain).

When cholecalciferol is absorbed, it is incorporated into chylomicrons and enters the lymphatic system before entering the bloodstream. Oral vitamin D seems to be less efficient than skin-synthesized vitamin D [90], maybe due to a higher retention in body fat. Calcifediol is more hydrophilic and, therefore, after ingestion, is absorbed into the venous portal system, immediately increasing circulating concentrations of 25(OH)D3, which is available within hours (Figure 6), to be a substrate for calcitriol synthesis in the kidney and extra-renal tissues, such as broncho-alveolar lung cells, immune cells, or other potential target tissues.

Calcifediol’s ease of absorption and availability is especially relevant in patients with severe fat malabsorption [89]. Furthermore, calcifediol does not require hepatic 25-hydroxylation, which is of great importance in clinical situations where rapid restoration of serum 25OHD is desirable and CYP2R1 expression is compromised.

CYP2R1 mutations are rare in the general population, and it seems that only bi-allelic mutations create problems in producing sufficient 25OHD [91]. The functional impairment of CYP2R1 activity, however, has been well demonstrated in several animal models of obesity, diabetes, or glucocorticoid excess [92] and is likely in patients with obesity or type 2 diabetes, with malabsorption [93] or with inflammatory lung diseases, such as COPD or asthma [94,95]. Thus, treatment with calcifediol is more effective than cholecalciferol in increasing serum 25OHD concentrations in these patients. An additional advantage of oral calcifediol is its more linear dose–response curve, whereas a higher intake of ergocalciferol/cholecalciferol results in a plateau effect [89]. This is relevant when rapidly elevated serum 25OHD levels are needed, as for example in patients with COVID-19.

Therefore, oral calcifediol is more potent than cholecalciferol, according to the results of nine RCTs, comparing physiological doses of oral cholecalciferol with oral calcifediol [86] and in clinical trials where the two drugs have been compared head-to-head [87].

The oral administration of 20 μg of calcifediol compared to 800 IU (20 μg) of cholecalcifeol was significantly more effective and faster in increasing serum 25OHD concentrations in postmenopausal women in the range above 30 ng/mL; furthermore, it produced significantly more pronounced suppression of eotaxin, IL-12, monocyte chemoattractant protein 1 MCP-1, and macrophage inflammatory protein 1 beta MIP-1β [96], which are markers implicated in the severity of COVID-19 [97].

Treatment with calcifediol prescribed for whatever health reason, such as osteoporosis, improves the 25OHD status [87,98], and thus may reduce the risk and impact of COVID-19 [53]. Indeed, in a retrospective cohort study in the Barcelona area (Spain) on a population of 4.6 million inhabitants collected in the public health system registries, from April 2019 to February 2020, the risk of COVID-19 infection during the first wave of the pandemic was assessed in patients who were prescribed cholecalciferol (*n* = 108,343) or calcifediol (*n* = 134,703) during the previous 4 months and were compared with propensity score-matched untreated controls [99]. In cholecalciferol-supplemented patients, the hazard ratio for infection was significantly lower (HR = 0.95; 95% CI: 0.91–0.98). Serum 25OHD levels were measured in a subpopulation of patients. Patients on calcifediol treatment, who achieved 25OHD levels above 30 ng/mL, suffered a lower rate of SARS-CoV-2 infection (HR = 0.69; 95% CI: 0.61–0.79), a lower risk of severe COVID-19 (HR = 0.61; 95% CI: 0.46–0.81) and lower risk of COVID-19 mortality (HR = 0.56; 95% CI: 0.42–0.76). These parameters were statistically significantly lower compared to untreated 25OHD-deficient patients (<20 ng/mL). Similarly, when patients had been supplemented with cholecalciferol, both the rate of SARS-CoV-2 infection (HR = 0.66; 95% CI: 0.57–0.77), the risk of severe COVID-19 (HR = 0.72; 95% CI: 0.52–1.00) and COVID-19 mortality (HR = 0.66; 95% CI: 0.46–0.93) were significantly lower. The same report also described a reduced risk of SARS-CoV2 infection and COVID-19 mortality in patients with stage 4–5 chronic renal failure treated with calcifediol [99]. These results are similar, although of a lesser magnitude, to those observed in a cohort of patients with COVID-19 treated with calcitriol [99].

Similarly, a study was carried out in another retrospective cohort of 15,968 patients, including all hospitalized for COVID-19 in Andalusia (Spain) between January and November 2020, obtained from the central registry of electronic medical records (Andalusian Population Health Database; BPS). The effect of the administration of vitamin D, or its metabolites, in the 15–30 days prior to hospitalization was assessed with respect to patient survival. Both Kaplan–Meier survival curves and hazard ratios supported an association between prescription of these metabolites and patient survival. The association was stronger for calcifediol (HR = 0.67; 95% CI: 0.50–0.91) than for cholecalciferol (HR = 0.75; 95% CI: 0.61–0.91). The relationship was maintained when a 30-day period before hospitalization was assessed but with a slightly lower effect (calcifediol (HR = 0.73; 95% CI: 0.57–0.95); cholecalciferol (HR = 0.88; 95% CI:0.75–1.03)), suggesting that the closer the treatment is to hospitalization, the greater the protective effects [100]. These results suggest that improving serum 25OHD concentration may improve the prognosis of COVID-19. Therefore, treatment with calcifediol in patients with COVID-19 could be of potential therapeutic benefit by improving 25OHD status more rapidly, and thus be immediately available in target cells, to combat the effects of SARS-CoV-2 in COVID-19 [8,53].

In order to investigate the potential therapeutic benefit of calcifediol, two intervention strategies have been designed. One using high doses of calcifediol [101,102,103], and the other using much lower doses [104]. The first published study, using a high-dose approach, was a parallel, open-label, randomized, double-masked pilot clinical trial conducted at the Hospital Universitario Reina Sofía in Córdoba (Spain) (preliminary to the clinical trial registered as “Prevention and treatment with Calcifediol of Coronavirus induced acute respiratory syndrome (SARS) COVID-19 (COVIDIOL)” [NCT0436690]) [101]. Thus, in 76 consecutive patients hospitalized with COVID-19, clinical pictures of acute respiratory infection were confirmed by a radiographic pattern of viral pneumonia and by positive PCR for SARS-CoV-2 with CURB65 severity scale (recommending hospital admission in case of total score > 1). All hospitalized patients received the best available treatment, the same standard of care, (according to hospital protocol), and a combination of hydroxychloroquine (400 mg every 12 h on the first day and 200 mg every 12 h for the next 5 days) and azithromycin (500 mg orally for 5 days).

**Figure 7:**
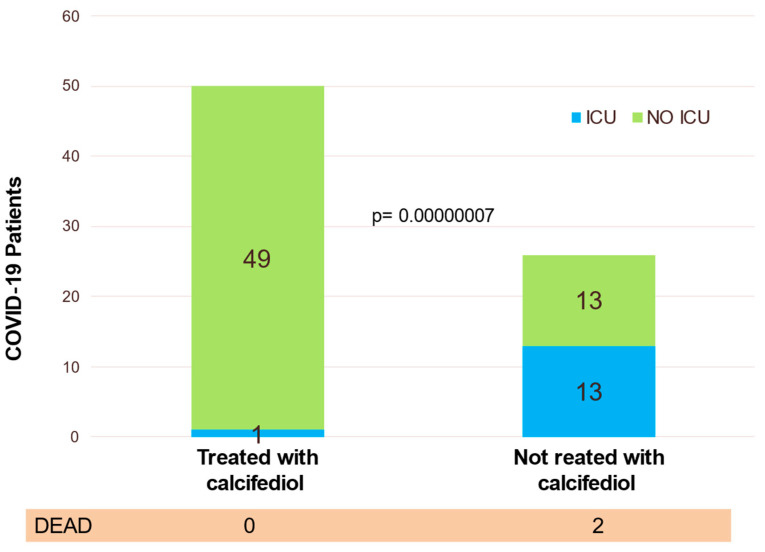
Effect of calcifediol treatment (0.532 mg on day 1 and 0.266 mg on days 3, 7, 14, 21, and 28). Parallel open label randomized double masked, double blinded, pilot clinical trial. Blue: ICU; yellow: no ICU. Elaborated from data obtained from Entrenas Castillo et al. [101].

Eligible patients were assigned to oral calcifediol/non-calcifediol in a 2:1 ratio, by electronic randomization. The treatment regimen, designed based on the kinetics of calcifediol in the formulation used (Figure 7), consisted of oral calcifediol (0.532 mg on the day of admission), followed by doses of 0.266 mg on days 3 and 7, and then weekly until discharge or admission to the intensive care unit (ICU). The results of effectiveness were compelling: of 50 patients treated with calcifediol, only one required ICU admission (2%), whereas, of 26 untreated patients, 13 required admission (50%) (*p* = 0.00000077). The estimated odds ratio of univariate risk for ICU in patients treated with calcifediol versus untreated with calcifediol: 0.02 (95% CI 0.002–0.17). The odds ratio of multivariate risk estimate for ICU in patients with calcifediol treatment vs. ICU without calcifediol treatment (adjusting for hypertension and type 2 diabetes): 0.03 (95% CI: 0.003–0.25). Of the patients treated with calcifediol, none died, and all were discharged without complications. The number of deaths was too small to achieve statistical significance against a null hypothesis of no effect, but the result is consistent with the plausible hypothesis that the decrease in mortality would be similar to the decrease in ICU admissions (Figure 7).

Shortly thereafter, a retrospective study of patients hospitalized for PCR-confirmed COVID-19 infection (excluding the patients involved in the just mentioned pilot study) addresses mortality reduction in patients treated with calcifediol [102]. Patients from five hospitals in Andalusia (Spain) (*n* = 537) hospitalized due to COVID-19 received standard care for pre-existing comorbidities and calcifediol or not, according to the treatment schedule of the pilot study cited above. Patients in one hospital received the option to receive calcifediol, while this option was not available in the other hospitals. Slightly more patients in the calcifediol-treated group had one or more comorbidities at baseline. In-hospital mortality during the first 30 days was 17.5%. The OR of death for patients receiving calcifediol (5% mortality rate) was 0.22 (95% CI 0.08–0.61), compared to patients not receiving calcifediol (20% mortality rate; *p* = 0.0005). In the multivariable logistic regression model, there was a significant difference in the mortality in patients who received calcifediol, compared to patients who did not (OR = 0.16; 95% CI 0.03–0.80) [102].

**Figure 8:**
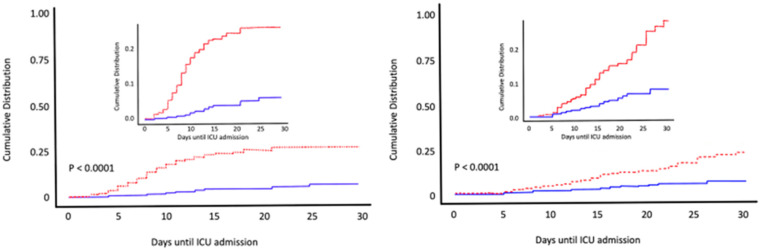
Early calcifediol administration and outcome of COVID-19. Patients (*n* = 838) hospitalized for COVID-19 received best available treatment and standard care for pre-existing comorbidities. Treatment groups were based on having received from admission (1) oral calcifediol (25OH D3) in soft gelatin capsules (0.532 mg), then oral calcifediol (0.266 mg) on days 3 and 7, then weekly until discharge or ICU admission (*n* = 447) represented in red; (2) no calcifediol treatment (*n* = 391) represented in blue. Cumulative distribution of patients presenting with ICU admission or in-hospital death according to treatment groups. Patients hospitalized with COVID-19 on calcifediol treatment, compared to those who did not receive calcifediol showed) a lower need for ICU admission (45% vs. 21%), reduced risk (OR 0.13, 95% CI 0.07–0.23) *p* < 0.0001 (**left**), and significantly lower in-hospital mortality during the first 30 days (7% vs. 15.9%,) OR 0,21 [95% CI 0.10–0.43 *p* < 0.0001) (**right**). Elaborated from data obtained from Nogues X et al. [103].

A larger observational cohort study included patients admitted to COVID-19 wards at Hospital del Mar; Barcelona (Spain) [103]. Calcifediol treatment significantly reduced the need for ICU support and significantly reduced mortality (Figure 8). Of 838 patients, 447 received calcifediol, while 391 were not treated on admission. The prescription of calcifediol was based on the ward to which they were assigned, depending on bed availability. In five of the eight wards patients received calcifediol, while in the other three wards they did not. Otherwise, treatment was similar and there were no significant differences in patient characteristics. Among those treated on admission with calcifediol, 4.5% required ICU admission, compared to 21% in the untreated group. The logistic regression of calcifediol treatment on ICU admission, adjusted for age, sex, baseline linearized 25OHD levels, and comorbidities showed that treated patients had a reduced risk of requiring ICU admission (OR 0.13, 95% CI 0.07–0.23). In addition, 7% of the 55 treated with calcifediol died on admission compared to 15.9% of the untreated. Adjusted results showed a reduced mortality risk with an OR 0.21 [95% CI 0.10, 0.10–0.43]) [103].

The first results of the daily low-dose calcifediol administration strategy, a randomized, double-blind, placebo-controlled, multicenter, clinical trial implemented by the Tehran University of Medical Sciences and Shahid Beheshti University of Medical Sciences (Iran) have just been published [104]. The trial included patients, admitted to the referral hospital for COVID-19, with serum 25OHD levels below 30 ng/mL. All patients received the same standard of care (a combination of hydroxychloroquine, azithromycin, and, in patients with pneumonia, ceftriaxone). Subjects in the treatment group (*n* = 53) received calcifediol, 25 μg administered orally once daily, and the nontreatment group (*n* = 53) received placebo. At one month of treatment, serum 25OHD levels were significantly increased in patients receiving calcifediol (42.0 ± 2.3 ng/mL) compared to the placebo group, 19.3 ± 1.7 ng/mL. After 60 days, 24 patients in the treatment group had levels of 59.6 ± 3.8 ng/mL vs. 19 patients in the placebo group 19.4 ± 1.6 ng/mL (*p* < 0.001). Treatment with oral calcifediol was associated with a significant increase in the percentage of lymphocytes and a decrease in the neutrophil-to-lymphocyte ratio in calcifediol-treated patients, with an overall lower trend for hospitalization, length of time in the intensive care unit, and need for respiratory support and mortality, but the differences were not statistically significant.

The big and main difference with the three previous studies was that the first week dose of calcifediol in the first three trials was 1.064 mg (0.532 in the first two days), with a high availability of 25OHD3 for use in target organs in the first few hours, compared to 0.175 mg in the Iranian study.

The available data strongly suggest that treatment with calcifediol can decrease the severity of COVID-19, as evidenced by the reduced need for intensive care and decreased mortality risks. It is a cost-effective treatment, free of major adverse effects and widely available, and could have positive implications for the treatment of the disease worldwide. However, we need the results of ongoing large, randomized trials to complete the evidence. In the meantime, based on the available data we recommend rapid correction of 25OHD deficiency in all COVID-19 subjects.

## 7. Conclusions

SARS-CoV2 infection has peculiarities that make the treatment of COVID-19 particularly complicated. The severe disease is characterized by an unbalanced host response to SARS-CoV-2, which, following intracellular viral replication, induces a reduction in innate antiviral defenses leading to the exuberant production of pro-inflammatory cytokines/chemokines, with inadequate recruitment of inflammatory populations of monocytes and macrophages with decreased cell surface expression of ACE2, thus losing a lung protective mechanism, leading to increased inflammation, oedema and more severe ARDS, and increased cardiovascular and multi-organ involvement, increasing the risk of thromboembolism. The intensity of these responses will determine the intensity of clinical outcomes in COVID-19.

From a mechanistic perspective, there are good reasons to postulate that stimulation of the VDR signaling pathway may have multiple functional actions in COVID-19: (1) in the early viral phase through innate antiviral effector mechanisms, including induction of antimicrobial peptides, such as cathelicidin and defensin and autophagy; (2) in the later hyperinflammatory phase of COVID-19 it may generate beneficial effects by decreasing the cytokine/chemokine storm, producing a shift from a Th1 and Th17 phenotype towards adaptive immune responses with an amplified Th2 phenotype; regulating the renin–angiotensin–bradykinin system (RAAS); modulating neutrophil activity and maintaining the integrity of the pulmonary epithelial barrier; stimulating epithelial repair; and directly and indirectly decreasing the increased coagulability and prothrombotic tendency associated with severe COVID-19 and its complications, including multiple organ fibrosis and probably minimizing post-COVID-19 syndrome.

Calcifediol provides pharmacokinetic advantages that give it a certain superiority for use in COVID-19. It is very hydrophilic and, therefore, after ingestion, is absorbed via the venous portal system and does not require hydroxylation at position 25, immediately increasing circulating concentrations of 25(OH)D3; it is available within hours, and in a stable manner, to be the substrate for calcitriol synthesis in bronchoalveolar lung cells, immune cells, or other potential target tissues in COVID-19.

The available data strongly and consistently suggest that treatment with calcifediol can reduce the severity of COVID-19, as evidenced by a reduced need for intensive care and a decreased risk of mortality. It is cost-effective, without significant adverse effects and widely available, and could have positive implications for the treatment of the disease worldwide. Of course, the evolution of COVID-19 is influenced by many other risk factors, such as age, gender, obesity, and nutritional factors, such as vitamin K have been suggested as disease modifiers of SARS-CoV-22 infection [105].

In conclusion, we, therefore, propose to consider using calcifediol at the doses described for the rapid correction of 25OHD deficiency in all patients in the early stages of the disease, in association, if necessary, with the new oral antiviral agents, such as molnupiravir, fluvoxamine, plitidepsin, paxlovid, etc. [106,107].

## Data Availability

Not applicable.
